# Supplementary material for: Regional disparities in antenatal care utilization in Indonesia
Source: PLoS One. 2020 Feb 13;15(2):e0224006. doi: 10.1371/journal.pone.0224006 (PMC7018075; doi:10.1371/journal.pone.0224006)
Supplement: Attachment — Submitted filename: Response to reviewers.docx [file pone.0224006.s001.docx]

Response to reviewers

Reviewer 1: I have incorporated all of your suggestions into my revisions. They were very helpful. Thank you. I included Socio-demographic analysis in binary logistic regression. This analysis will provide clear guidance for policymakers of clear targets.

Reviewer 2: I have incorporated all of your suggestions into my revisions. They were very helpful. Thank you
